# Simultaneous, Bilateral Acute Subscapularis Ruptures and Their Arthroscopic Management

**DOI:** 10.1155/2019/7964351

**Published:** 2019-04-28

**Authors:** Andrew M. Schwartz, Jacob M. Wilson, Kyle Hammond

**Affiliations:** Department of Orthopaedics, Emory University School of Medicine, Atlanta, GA, USA

## Abstract

We present the first known case of bilateral, acute ruptures of the subscapularis tendons following a bicycle accident in a 43-year-old male. He underwent right shoulder arthroscopic, anatomic subscapularis tendon repair two weeks postinjury, with the left side staged for surgical treatment six weeks after the index procedure. Postoperatively, the patient remained in a sling for 6 weeks before advancing with therapy protocols. The interval between arthroscopic treatments allowed for independence with activities of daily living and focused, early therapy for each shoulder. This approach yielded a right-sided constant score of 89 and subjective shoulder value of 90%; the left side was 87 and 90%, respectively, at 33 months postoperatively. The patient's only postoperative complaint was slightly diminished external rotation, a near-universal limitation after unilateral repair. This represents a successful outcome that balances functional independence, concentrated rehabilitation, and adherence to safe indications for primary repair. While bilateral traumatic shoulder injuries in a young person is a rare clinical entity, early and staged treatment can lead to good patient outcomes.

## 1. Introduction

Simultaneous, bilateral acute shoulder injury is a rare phenomenon often attributed to electric trauma or generalized seizure activity. Classically, this results in dislocation with or without associated fracture or soft tissue injury [1–5]. The rarity of this injury pattern is likely owed to the unlikely occurrence of the required mechanism of injury (direct blow to both shoulders or a fall onto two outstretched arms). However, in the nontraumatic setting, bilateral rotator cuff (RC) tears are relatively common and typically atraumatic and degenerative in nature. In fact, bilateral RC tears occur in half of patients by 66 years of age, and patients with bilateral ruptures average nine years older than patients with unilateral ruptures [6]. In these clinical scenarios, rotator cuff symptoms are often a function of age [6]. Unlike traumatic tears, these are typically partial tears and the degree of tendon injury correlates with functional impact and pain [6]. Previous works regarding bilateral RC repairs have all been conducted in patients with chronic tears [7].

When considering rotator cuff tears, subscapularis tears are particularly challenging as the subscapularis is the primary internal rotator at the shoulder, a motion that is integral in nearly all activities of daily living (ADLs). As such, full thickness tears are generally indicated for surgery [8, 9]. While a thorough physical exam is important, the sensitivity and tear size correlation of clinical exam are inferior to the sensitivity of MRI (100% in full thickness tears) [10]. Further, the reported sensitivity of exam maneuvers is variable in the literature, with Naimark et al. reporting a 61% sensitivity with the belly-press maneuver and 63% with the lift-off test [10]; Barth et al. suggest a superior sensitivity with the bear-hug maneuver (60%), while other special exams had only 40% sensitivity [11]. Nonetheless, isolated rotator cuff injury to the subscapularis is a rare occurrence that necessitates high clinical suspicion, particularly in the setting of the classic forced abduction-external rotation injury mechanism [8, 12, 13].

While subscapularis tears require expeditious surgery to avoid retraction, atrophy, and subsequent functional limitation, they remain rare with an incidence of just over 1% [13]. Bilateral traumatic shoulder injuries are also uncommon clinical entities and have potentially devastating functional impacts. Nonoperative treatment of traumatic tears may lead to fatty degeneration, diminished function, and loss of strength, which would render useful upper extremity tasks impossible. In this report, we present a case of traumatic, bilateral full-thickness subscapularis tendon ruptures and the subsequent staged, arthroscopic surgical management.

## 2. Case

**Figure 1:**
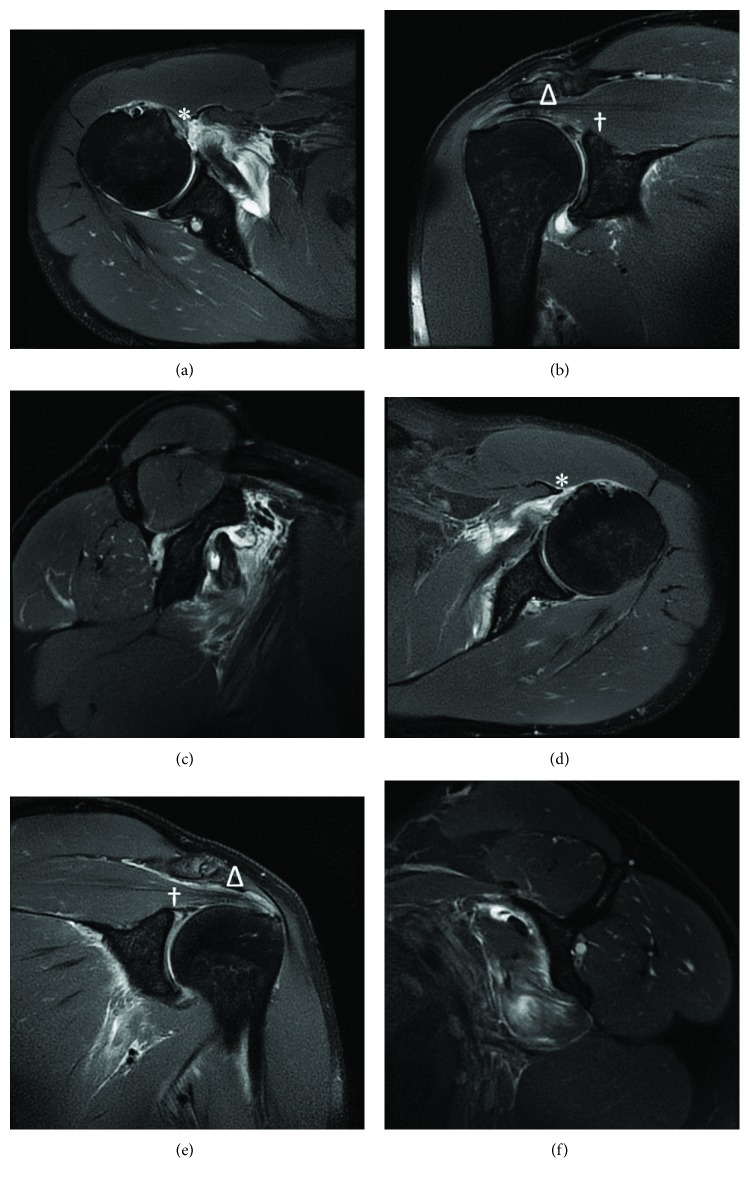
Noncontrast MRI of (a) axial, (b) coronal, and (c) sagittal views of the right shoulder; (d) axial, (e) coronal, and (f) sagittal views of the left shoulder. ∗ denotes a torn subscapularis tendon. Δ denotes an intact supraspinatus tendon. † denotes a type 4 SLAP tear. Sagittal views demonstrate edematous changes and retraction, without significant fatty infiltration.

**Figure 2:**
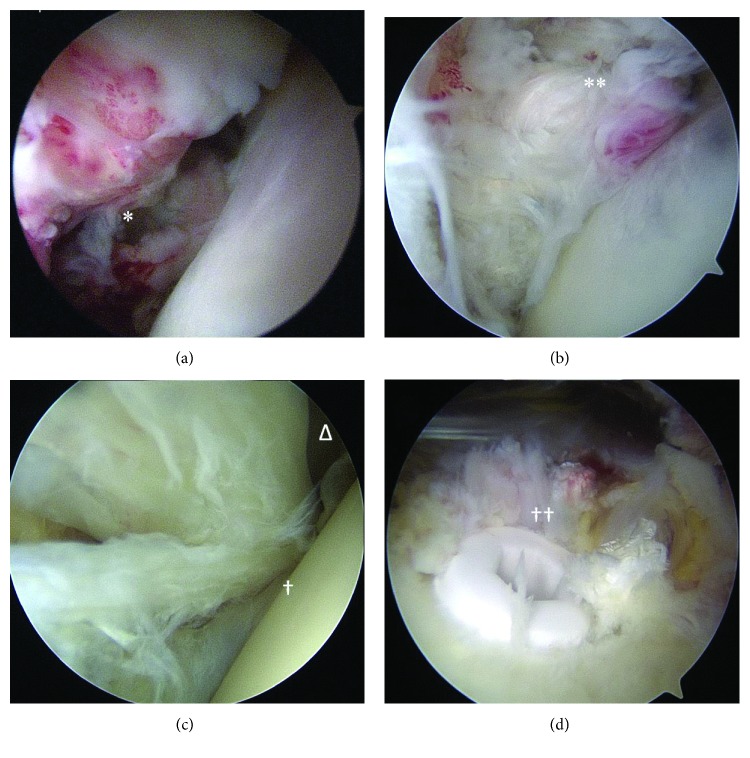
Intraoperative arthroscopic photographs of the right shoulder; left shoulder images were symmetric. All captured from standard posterior portal. (a) ∗ denotes torn subscapularis tendon. (b) ∗∗ denotes repaired subscapularis tendon. (c) Δ and † denote a torn long head of the biceps tendon. Δ denotes a type 4 SLAP tear. (d) †† denotes an arthroscopic long head of the biceps tenodesis.

The patient is a 43-year-old male with a history of a traumatic left distal biceps tendon rupture (now, three years status post uncomplicated repair), remote right shoulder pain managed successfully with physical therapy without recurrence, and chronically low testosterone managed with weekly testosterone injections—who presented one day after a traumatic bilateral shoulder injury. The patient describes an ejection over the handlebars of his bicycle, landing in a “push-up” position, shoulders abducted to approximately 90°, and elbows flexed to 90°. He noted deep shoulder pain and internal rotation limitations, bilaterally. At the time of presentation, his pain had mildly improved, but functional status of both shoulders remained unchanged. On exam, his left shoulder was tender to palpation anteriorly with passive forward flexion to 140° and 4/5 strength, passive abduction to 120° and 4/5 strength, external rotation to 20° and 4/5 strength, internal rotation to 10° and 3/5 strength, a positive liftoff test, positive bear hug test, and a positive belly press test. His right shoulder was tender to palpation anteriorly with passive forward flexion to 125° and 4/5 strength, passive abduction to 90° and 4/5 strength, external rotation to 20° and 4/5 strength, internal rotation to 5° and 2/5 strength, a positive lift-off test, and a positive belly press test. X-rays of both shoulders were obtained with anteroposterior (AP), scapular-Y, and axillary views, which showed no signs of fracture, dislocation, or deformity, bilaterally. A noncontrasted MRI (Figure 1) obtained two days later demonstrated complete rupture of the right subscapularis tendon, just distal to the musculotendinous junction; complete rupture of the left subscapularis tendon, just distal to the musculotendinous junction; and bilateral type 4 SLAP lesions. There were no other signs of osseous or soft tissue injury; the remainder of both rotator cuffs was intact, and there was no fatty infiltrate in either subscapularis muscle belly. The patient was reevaluated in the office 10 days postinjury, and a video was obtained of his preoperative examination (Video 1), at which point he was scheduled for arthroscopic subscapularis tendon repair of the right shoulder (2 weeks postinjury), as this was his dominant side, and then the left shoulder seven weeks later. Intraoperative findings (Figure 2) included complete subscapularis rupture on both sides, with type 4 SLAP lesions and associated partial tear of the long head of the biceps at its labral origin. As suggested on MRIs, the remainder of the rotator cuffs was intact. Given the delayed nature of the left-sided surgery, there were some muscle retraction and scarring of the left subscapularis that increased the difficulty of surgery, though both tendons were adequately repaired without need for soft tissue grafting or augment.

## 3. Surgical Technique

The surgeries were both performed by the same surgeon (KEH) and both performed with similar techniques. The beach chair position was used, as well as regional and general anesthesia. The initial portals were standard posterior and anterior portals to view the glenohumeral joint. Within the joints of both shoulders, we visualized similar pathologies related to the superior glenohumeral ligaments, superior labrum (Figure 2(c)), and subscapularis tendons (Figure 2(a))—where all these structures were torn and retracted from their origins and the biceps tendon was in its anatomic groove. The subscapularis tendon tears were noted to be complete, without glenohumeral joint instability, which can be classified as Lafosse type IV tears [14]. The remaining anatomic features within the glenohumeral joint and rotator cuff were within normal limits. Once a limited debridement of the joint was concluded, we placed a #2 nonabsorbable suture within the biceps tendon about 6 mm from the superior glenoid anchor. A biceps tenotomy was then performed at the anchor, and then, the bicipital stump was debrided to remove unstable tissue from the superior labrum to create a smooth contour. We utilized a stitch within the biceps tendon, in preparation for a biceps tenodesis. We then could directly visualize the “comma” sign [15] to identify the subscapularis tendon avulsion through the torn coracohumeral/superior glenohumeral ligaments, as well as the bare footprint of the lesser tuberosity. Leaving the scope in the posterior portal, we utilized the anterior and an accessory biceps tenodesis portal to prepare the lesser tuberosity footprint and place the #2 nonabsorbable sutures within the substance of the subscapularis tendon. Two 4.75 mm biocomposite suture anchors were then placed within the proximal and distal aspects of the medial ridge of the bicipital groove, which allowed the subscapularis footprint to be completely covered with the repaired tendon, without tension (Figure 2(b)). The scope was then switched into the subacromial space from the posterior portal. Within the subacromial space, there were signs of bursitis and abrasions to the coracoacromial ligament. With these findings and his history of rotator cuff impingement, we elected to perform a debridement and subacromial decompression. We then visualized down the anterior humerus after moving the scope into the lateral portal. As the biceps tendon was localized within the bicipital groove, we then decompressed the groove and released the transverse humeral ligament. An anterior accessory portal was then made to drill a 7.5 mm hole within the bicipital groove, and then, under anatomic tension, we placed a 7 mm biocomposite tenodesis screw with the biceps tendon into the humerus, just proximal to the pectoralis major tendon. The use of anatomic landmarks for optimal tension of biceps tenodesis is a similar protocol as described by Neviaser et al. [16]. The residual biceps tendon proximal to the tenodesis site was excised from the shoulder. This completed the biceps tenodesis (Figure 2(d)). The left shoulder surgical procedure was performed in the exact same fashion, with the same fixation methods and had the same arthroscopic findings. The second surgery was performed 7 weeks after the first surgery and 9 weeks postinjury. It is worth noting that we considered the potential for diminished healing potential by delaying treatment of the contralateral side, but evidence against delayed treatment has been limited to histologic inquiry without clinical correlation at this point [17]. Clinical data suggests better outcomes after repair within 6 months [18]. As such, we elected to proceed with the staged protocol to facilitate focused physical therapy and contralateral extremity independence in the early postoperative periods.

## 4. Postoperative Protocol

The patient began therapy on the right upper extremity immediately following surgery to optimize function of his right arm in preparation for his left-sided surgery. Between surgeries, the patient was encouraged to use his preoperative left upper extremity as tolerated to allow for ADLs. Postoperatively, each shoulder went through a similar protocol; both shoulders were immobilized in slings in internal rotation for 6 weeks and followed a standard rotator cuff repair rehabilitation protocol. There were specific limitations placed upon the patient, related to the subscapularis. We did not allow any external rotation beyond neutral for the first 4 weeks. After the 4^th^ week, we allowed the therapy program to progress the patient's external rotation 10 degrees each week, and at week 12, there was unlimited range of motion in all passive plans. Active-assisted range of motion was begun at 8 weeks and light resistance training began at week 12. As he was progressing quickly and adequately with his recovery, we allowed all activities as tolerated and released him for full activity at week 22.

Our follow-up care occurred at the following intervals for each shoulder: 1 week postoperatively, 6 weeks, 12 weeks, 26 weeks, and around 1 year postoperatively. At 14 months from his left shoulder surgery and 16 months from his right shoulder follow-up, he was pain-free and had returned to all activities, including working, bicycling, and weightlifting without any limitation. His only complaint was bilateral tightness in external rotation beyond 90° of external rotation, while his arm is abducted. His left shoulder ROM at terminal follow-up was notable for neutral internal rotation that allowed him to reach the level of L2 on his back, neutral external rotation to 40°, external rotation in 90° of abduction to 85°, and internal rotation in 90° to 60°. His right shoulder ROM was neutral internal rotation that allowed him to reach the level of L3 on his back, neutral external rotation to 35°, external rotation in 90° of abduction to 85°, and internal rotation in 90° to 60°. Forward flexion and abduction were full, to 180°, bilaterally. He had 5/5 strength in all directions, bilaterally. At final follow-up, constant scores were obtained which were 89 for the right shoulder and 87 for the left shoulder. Subjective shoulder values were 90% for both shoulders.

## 5. Discussion

We reported a unique case of acute simultaneous, bilateral subscapularis tendon ruptures, which in this case was the result of a fall off a mountain bike. While this presents a challenging clinical scenario, our report demonstrates one management option which achieved favorable outcome in our patient, performed arthroscopically as an outpatient.

Of the rotator cuff muscles, the subscapularis muscle has the most mass and strength. Consequently, tears of the tendon are more commonly an insidious process resulting from chronic wear or impingment [13]. While chronic subscapularis tears are relatively common, they are usually associated with additional RC pathology [13]. This patient had no preexisting pain or functional limitation prior to his fall, which resulted in both acute pain and shoulder rotational deficits, consistent with his bilateral acute subscapularis injuries. However, he did have a history of traumatic distal biceps tear and is an active testosterone replacement therapy user. Even when used appropriately, testosterone is associated with a fibrosing effect and decreased elasticity in tendons and has been associated with supraspinatus tendinopathy [19–22]. As such, while both the biceps tendon injury and the current bilateral subscapularis tendon injuries were the result of traumatic events, the threshold for tendon injury may have been lowered by testosterone replacement therapy. One study found that while the overall prevalence of subscapularis tendon ruptures was 37%, only 1.4% of subscapularis tears occurred as isolated injuries [23]. Associated proximal bicipital pathology such as groove dislocation and tendon degeneration is also common and occurs concurrently as frequently as 56% of the time [24]. The rarity of subscapularis tear in the absence of a supraspinatus tear is likely due, at least in part, to the interdigitation of portions of both the subscapularis and supraspinatus tendons [12].

Acute, traumatic subscapularis tears, in contrast to degenerative tears, are rare. These injuries are mechanistically the result of forced hyperextension or external rotation moment applied to the abducted shoulder—owing to the muscle's orientation [8, 12, 25]. Like our patient, acute tears tend to occur in younger patients [8]. Our patient's landing position, the “push-up” position, when he was thrown from his motorcycle likely had mixed components of forced shoulder extension and external rotation on impact with the ground. His exam was consistent with the findings expected in a subscapularis rupture; internal rotation strength is decreased but not absent as there is residual function from the pectoralis major, latissimus dorsi, and teres major [12].

While, to our knowledge, this case possibly represents the first published incident of bilateral subscapularis traumatic ruptures, published evidence of simultaneous bilateral shoulder injury of any etiology is also scarce. The overwhelming majority of literatures on bilateral traumatic injuries are discussions of fracture-dislocation injuries as a result of forced contraction (electrocution, seizure, and one incident of mechanical trauma) [1–5]. These injuries are often debilitating, with osseous, chondral, and soft tissue damage necessitating arthroplasty reconstruction. Bilateral RC deficiencies are limited to small case series of degenerative tears that were indicated for surgery [26]. To that end, our patient appears to represent not only a clinically challenging case but also a case that is exceedingly uncommon.

Surgical repair of a traumatic RC injury is typically indicated to return the patient to functionality [12]. The decision to proceed with surgery for this patient required careful planning. The patient preferred that both shoulders be surgically addressed simultaneously. However, attention to ADLs must be considered. A 3-6-week bracing period and six weeks of obligate external rotation limitation are crucial in the early postoperative period to preserve the tendon repair [9]. As such, a unilaterally injured patient is substantially impaired in his or her ability to transfer, feed, engage in hygienic activities, and use the bathroom after repair. A simultaneous bilateral repair would result in obligate dependence for basic functions. Additionally, while surgical interventions for subscapularis tears have good or excellent results in greater than 90% of patients [27], arthroscopic repair performed within six months of injury is thought to have better outcomes [18]. To that end, we advocate for staged repair (i.e., one shoulder addressed at a time), with an interval of at least six weeks, to facilitate patient independence and postoperative protocol adherence in the early postoperative period.

Long-term outcomes for rotator cuff repair are dependent upon a patient's ability to return to work and physical activity, as well as strength and ROM exam. Namdari et al. used a ten-activity assessment of ROM and strength to quantify the minimal range of motion needed to successfully navigate ADLs [28]. The data suggested that the average patient needs 59° of external rotation with the shoulder abducted to 90° and 102° of internal rotation (at neutral shoulder abduction) which our patient was able to attain. Therefore, given our patient's maintained ROM and satisfaction with his functional status, we feel this case represents a successful treatment in a complex case, while utilizing an arthroscopic technique. While there is a paucity of literature on the management of traumatic bilateral subscapularis injuries, we believe that our staged approach optimized the patient's outcomes while preserving independence with ADLs and therefore adherence to postoperative protocol adherence, in the perioperative period. This protocol is therefore recommended for the management of nonemergent bilateral shoulder injuries indicated for operative intervention, as an arthroscopic staged procedure.

## Supplementary Materials

Supplementary Materials Supplementary Video: presenting examination of the patient presented in this case. The patient has full forward flexion and abduction but has limited internal rotation. He has limited internal rotation strength bilaterally. There is a positive belly press, bear hug, and lift-off test bilaterally.
